# Impact of the COVID-19 Pandemic on Medical Grand Rounds Attendance: Comparison of In-Person and Remote Conferences

**DOI:** 10.2196/43705

**Published:** 2024-01-03

**Authors:** Ken Monahan, Edward Gould, Todd Rice, Patty Wright, Eduard Vasilevskis, Frank Harrell, Monique Drago, Sarah Mitchell

**Affiliations:** 1 Vanderbilt University Medical Center Nashville, TN United States

**Keywords:** continuing medical education, COVID-19, distance education, professional development, virtual learning

## Abstract

**Background:**

Many academic medical centers transitioned from in-person to remote conferences due to the COVID-19 pandemic, but the impact on faculty attendance is unknown.

**Objective:**

This study aims to evaluate changes in attendance at medical grand rounds (MGR) following the transition from an in-person to remote format and as a function of the COVID-19 census at Vanderbilt Medical Center.

**Methods:**

We obtained the faculty attendee characteristics from Department of Medicine records. Attendance was recorded using a SMS text message–based system. The daily COVID-19 census was recorded independently by hospital administration. The main attendance metric was the proportion of eligible faculty that attended each MGR. Comparisons were made for the entire cohort and for individual faculty.

**Results:**

The observation period was from March 2019 to June 2021 and included 101 MGR conferences with more than 600 eligible faculty. Overall attendance was unchanged during the in-person and remote formats (12,536/25,808, 48.6% vs 16,727/32,680, 51.2%; *P*=.44) and did not change significantly during a surge in the COVID-19 census. Individual faculty members attendance rates varied widely. Absolute differences between formats were less than –20% or greater than 20% for one-third (160/476, 33.6%) of faculty. Pulmonary or critical care faculty attendance increased during the remote format compared to in person (1450/2616, 55.4% vs 1004/2045, 49.1%; *P*<.001). A cloud-based digital archive of MGR lectures was accessed by <1% of faculty per conference.

**Conclusions:**

Overall faculty attendance at MGR did not change following the transition to a remote format, regardless of the COVID-19 census, but individual attendance habits fluctuated in a bidirectional manner. Incentivizing the use of a digital archive may represent an opportunity to increase faculty consumption of MGR.

## Introduction

Medical grand rounds (MGR) has evolved from the bedside [1] to a weekly presentation to the entire department [2]. Due to the COVID-19 pandemic, the format of MGR has undergone another transition, from in person to remote. While MGR attendance patterns for in-person conferences have been reported [3], the impact of remote conferences on faculty attendance at MGR is unknown. The analysis of remote surgical conferences [4,5] has been limited by sample size and aggregate data.

We propose that including more faculty from multiple specialties and individual conference or attendee data will provide more robust analysis that may inform returning to an in-person format, maintaining a remote format, or using a hybrid approach. Therefore, using our institution’s cloud-based attendance recording database, we (1) evaluated MGR attendance over time before and after the transition to the remote format and (2) assessed the temporal relationship between our institution’s COVID-19 census and attendance at MGR conferences.

## Methods

### Study Design, Participants, and Setting

We performed a retrospective cohort study of MGR attendance for all Department of Medicine (DOM) clinical faculty at Vanderbilt Medical Center active between March 2019 and June 2021. All conferences before March 12, 2020, were in person, and all conferences on or following this date were remote.

### Attendee Characteristics

For each division within the DOM, the number of faculty eligible to attend each conference as well as the number of faculty that attended each conference were available, as was each faculty member’s academic rank (assistant, associate, or full professor).

### Recording of Conference Attendance

Attendance was recorded by a cloud-based continuing medical education (CME) system during the entire observation period. Faculty indicate their attendance by sending an SMS text message containing the unique numeric code for that conference to a specific CME number. Conference attendance is registered as a binary outcome. The number of faculty considered to have attended a conference was obtained directly from this system. The number of faculty considered not to have attended was defined as the difference between the number of faculty eligible to attend and the number for whom attendance was recorded. The proportion of attendance was defined as the ratio of those who attended to those who were eligible over a given time frame (ie, in person or remote).

### Individual-Level Attendance Data

For each faculty member, the CME system generates a unique user number that is not related to any other identification mechanism or coupled to any other database. By removing all identifying information from faculty members’ attendance data except this user number, we could track individual attendance over time without the capability of linking these data to a given faculty member’s actual identity.

### Archived Conferences

Beginning in November 2019, digital recordings became available shortly after each MGR. Attendance credit was not given for consuming MGR in this manner. The number of faculty members that accessed a given MGR and the date on which each faculty member accessed the conference were available from the archive.

### Acquisition of COVID-19–Related Data

Our institution tracked the census of hospital inpatients with positive COVID-19 tests as well as the subset of that group that required intensive care unit (ICU) care or mechanical ventilation. The COVID-19 burden on a given day included the total number of COVID-19 patients (cases) relative to the peak observed during the observation period (calculated as cases or peak), the proportion of patients with COVID-19 requiring ICU care relative to the number of cases (calculated as ICU or cases), and the proportion of patients with COVID-19 requiring mechanical ventilation (calculated as ventilator or cases). We defined the “surge” as the interval between December 2020 and January 2021, when COVID-19 cases were at their maximum.

### Statistical Analysis

The main analyses compared the attendance rates during the entire in-person and remote periods as well as during the surge. Additional analyses stratified attendance by academic rank. All comparisons were made using the chi-square test in GraphPad Prism (version 9.2.0; GraphPad Software). For individual attendees, the difference between attendance rates at in-person and remote conferences was calculated, as were the characteristics of the resulting distribution.

### Ethical Considerations

This investigation was considered nonresearch activity by the Vanderbilt Medical Center’s institutional review board (number 211362). The need for informed consent was waived because of the retrospective nature of the study.

## Results

### Cohort Characteristics and Overall Attendance Observations

Characteristics of the MGR conferences, speakers, and faculty attendees are displayed in Table 1.

**Table 1:** Conference and attendee characteristics.

Characteristics		Value	Value at the end of the observation (range during observation period)
**Conferences, n**			
	Total during observation period	101	N/A^a^
	In person (prepandemic)	47	N/A
	Remote (intrapandemic)	54	N/A
**Topic, n**			
	Cardiology	19	N/A
	Endocrine	10	N/A
	Gastroenterology	12	N/A
	General internal medicine	15	N/A
	Geriatric medicine	3	N/A
	Hematology or oncology	10	N/A
	Infectious disease	10	N/A
	Nephrology	7	N/A
	Pulmonary or critical care	7	N/A
	Rheumatology	5	N/A
**Speaker, n**			
	Internal	41	N/A
	External	60	N/A
**Faculty attendance^b^, mean (SD)**			
	Total eligible to attend	579 (22)	611 (544-612)
	Cardiology	100 (2)	103 (95-103)
	Endocrine	25 (2)	28 (23-28)
	Gastroenterology	41 (2)	43 (38-43)
	General internal medicine	175 (8)	187 (161-187)
	Hematology or oncology	65 (2)	69 (60-69)
	Infectious disease	43 (1)	45 (40-45)
	Nephrology	33 (2)	36 (31-36)
	Pulmonary or critical care	46 (2)	46 (42-49)
	Rheumatology	22 (1)	23 (21-23)
	Assistant professor	328 (16)	349 (279-350)
	Associate professor	107 (1)	109 (105-109)
	Full professor	143 (11)	149 (107-151)

^a^N/A: not applicable.

^b^The number of faculty in the subspecialties is fewer than the total due to not listing smaller divisions. Faculty categorized by academic rank may not sum to the total due to a small number of transitions between ranks.

**Figure 1:**
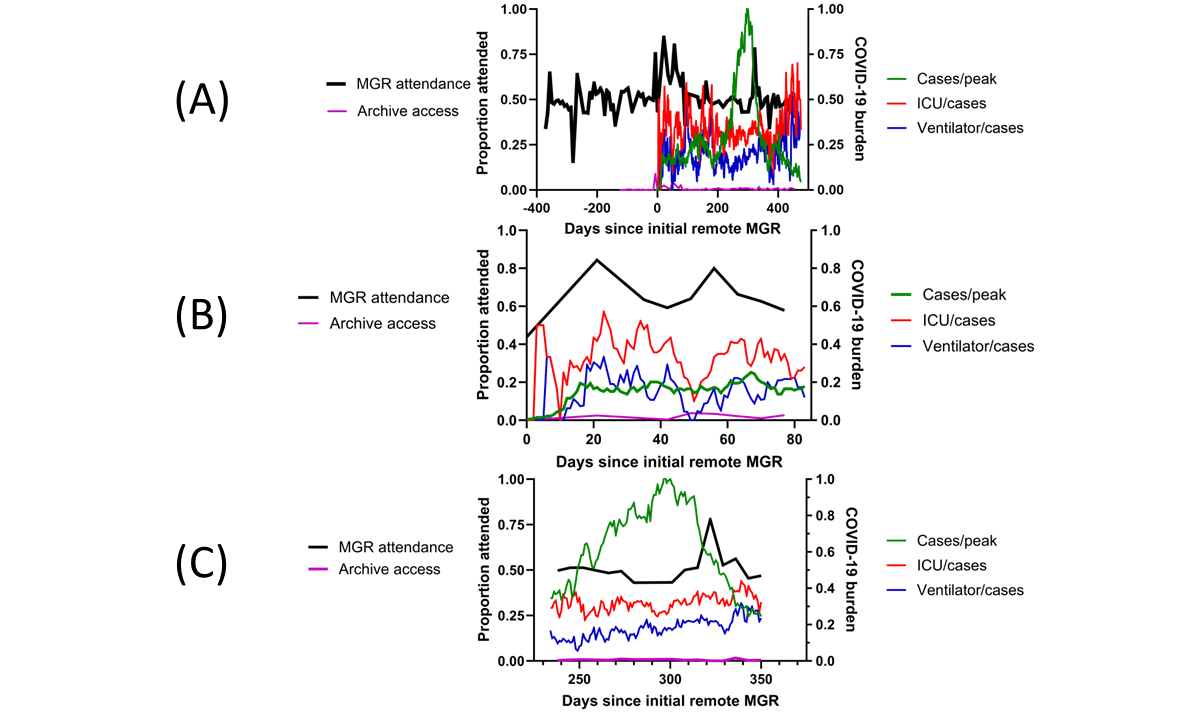
Time series of medical grand rounds (MGR) attendance and concurrent COVID-19 burden. (A) The entire observation period, (B) focus on the beginning of the remote format, and (C) focus on the surge. At the onset of the remote format, there is a nonsustained increase in attendance. As the COVID-19 census increased rapidly leading up to the peak census, there was no change in attendance. During the peak of the surge, there is a very small transient reduction in attendance followed by an extremely brief increase in attendance during a period of rapid decline in the COVID-19 census. Access to archived MGR lectures remained low during the entire observation period. ICU: intensive care unit.

Figure 1A shows (1) the time series of MGR attendance over the entire observation period and the number of times a given MGR was accessed from the cloud-based archive within 1 month of the conference, (2) the concurrent time series of COVID-19 cases as a proportion of the peak number recorded during the observation period, and (3) the time series of COVID-19 cases requiring ICU care and ICU cases requiring mechanical ventilation, both as proportions of the number of COVID-19 cases. Despite increases in remote attendance during the beginning of the pandemic (Figure 1B) and a brief increase as the surge began to subside (Figure 1C), there was no difference in attendance at MGR during the in-person format and the remote format over the entire observation period (12,536/25,808, 48.6% vs 16,727/32,680, 51.2%; *P*=.44). The proportion of faculty accessing the MGR digital archive remained low throughout the observation period, never exceeding 5% for any lecture and often not exceeding 1% (mean 0.7%, SD 1.3%).

**Figure 2:**
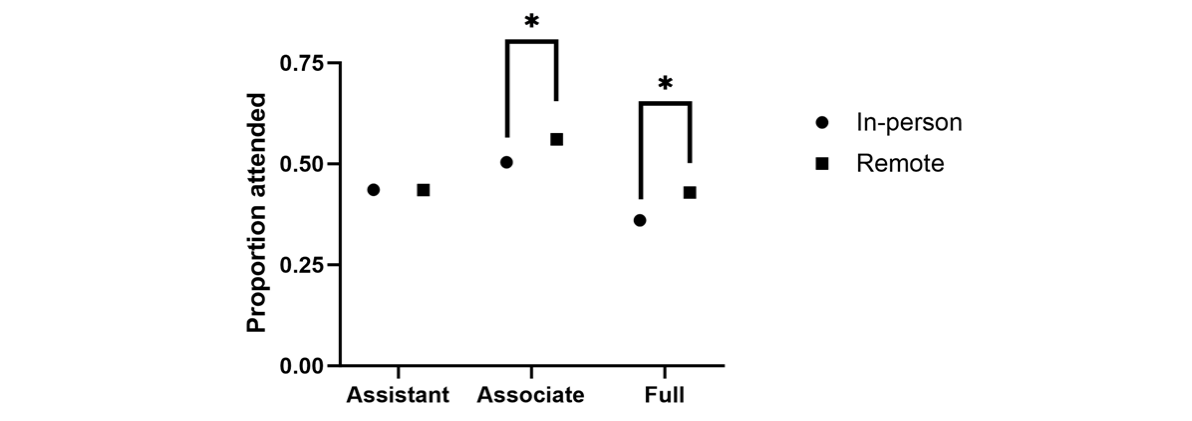
Attendance at medical grand rounds stratified by academic rank. Assistant professor attendance was the same regardless of conference format, whereas associate and full professor attendance increased during the remote format relative to in person. *P<.001.

MGR attendance stratified by academic rank across the in-person and remote formats is shown in Figure 2. Associate (3249/5788, 56.1% vs 2515/4989, 50.4%; *P*<.001) and full professor (3309/7718, 42.9% vs 2433/6757, 36%; *P*<.001) attendance was higher at MGR during the remote format relative to the in-person format.

### Subinterval and Subgroup Analyses

There was no difference in attendance during the surge compared to the 2 months before (October to November 2020; 2071/4218, 49.1% vs 2194/4229, 51.9%; *P*=.38) or 1 year before (December 2019 to January 2020; 2028/3990, 50.8% vs 2194/4229, 51.9%; *P*=.34).

**Figure 3:**
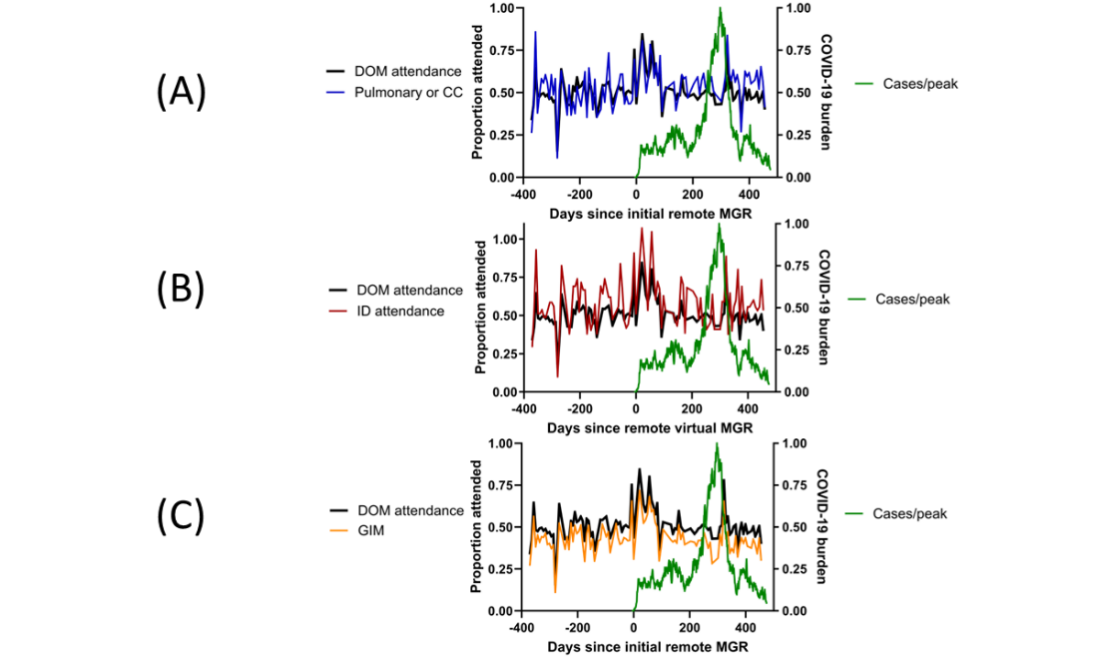
Selected subspecialty attendance trends. There are distinct qualitative patterns of medical grand rounds (MGR) attendance relative to the entire Department of Medicine (DOM) cohort for faculty in (A) pulmonary or critical care (CC), (B) infectious diseases (ID), and (C) general internal medicine (GIM).

The attendance trends of DOM subspecialties that were particularly impacted by the pandemic are superimposed on the overall DOM trend in Figure 3 for pulmonary or critical care (CC), infectious diseases (ID), and general internal medicine (GIM).

Pulmonary or CC attendance during the remote format was higher than during the in-person format (1450/2616, 55.4% vs 1004/2045, 49.1%; *P*<.001). This attendance pattern persisted while cases were rising and peaking during the surge, when demands on this portion of the faculty were likely greater than prepandemic. ID faculty had higher attendance throughout the entire observation period relative to the whole DOM cohort. The GIM faculty consistently attended MGR less frequently than the rest of the DOM cohort, including a sizable decrease during the peak of the surge.

### Individual-Level Analyses

**Figure 4:**
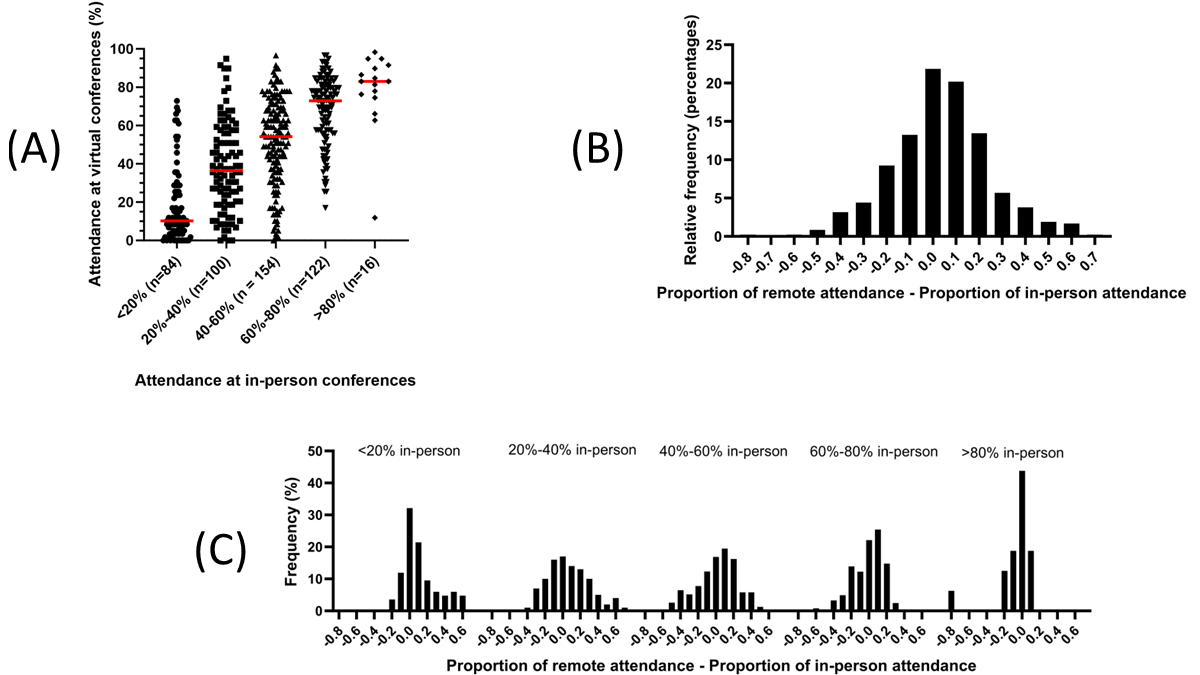
Individual-level attendance at in-person and remote medical grand rounds (MGR). (A) For a given level of attendance at in-person MGR, individual faculty member attendance at remote MGR fluctuated widely. (B) The distribution of the difference in attendance rates between conference formats for the entire cohort is relatively symmetric around the null, as expected given the overall lack of change. Nonetheless, extreme values of changes in attendance at the tails are noted. (C) Faculty that attended in-person MGR less frequently generally increased their attendance at remote MGR, while the opposite response was observed for those that frequently attended in-person MGR. Red bars indicate the mean.

Data were available for 476 faculty eligible to attend all the MGR during the observation period. As shown in Figure 4A, attendance rates during in-person conferences did not predict attendance rates for remote conferences. As displayed in Figure 4B, the distribution of the absolute difference between remote and in-person attendance rates is relatively symmetric around the null, but outliers at both tails are noted. Attendance decreased by at least 20% for nearly 15% (64/476; 13.4%) of faculty and increased by at least that amount for 20.2% (96/476) of faculty. The distribution of the differences in individual faculty attendance between remote and in-person conferences is shown in Figure 4C, stratified by in-person attendance rates. The distributions of the 2 lowest categories of in-person attendance exhibit positive skewness, while the remaining categories demonstrate negative skewness, indicating that the direction of the changes in individual attendance patterns observed with the transition in conference format varied based on in-person attendance. Lastly, 4.8% (23/476) of faculty exhibited absolute differences of 50% in attendance between formats.

## Discussion

### Principal Findings

Overall faculty attendance at MGR remained constant regardless of conference format, suggesting no disadvantage to the remote format. In addition, there may be substantial cost savings [6] and beneficial environmental impacts [7] associated with the remote format as it pertains to external speakers, who comprised the majority (60/101, 59.4%) of this cohort.

The increase in attendance of associate and full professors during the remote format may indicate fewer concurrent clinical obligations for these groups compared to their more junior colleagues. COVID-19–related MGR lectures at the beginning of the remote period may have led to the concurrent initial increase in attendance [8], but attendance quickly regressed to the mean, which was maintained even during a subsequent period of rapid rise and peak in COVID-19 burden.

Paradoxically, pulmonary or CC faculty attendance increased during the pandemic. It is possible that the attendance of the subgroup of non-ICU providers within pulmonary or CC may have increased during the pandemic while the attendance of their ICU-based colleagues declined. We speculate that the decreased attendance of the division of GIM was contributed to by lower attendance within the section of hospital medicine, perhaps because of burnout [9].

Individual faculty attendance habits did not remain static in response to the change in conference format. The pandemic or the remote format may have motivated faculty to attend MGR who did not regularly do so, thus taking the place of faculty that were unable to attend due to increased clinical or administrative responsibilities. The presence of outliers at both extremes of attendance shifts may enrich further investigations of specific drivers of conference attendance, which could inform decisions regarding conference format moving forward.

Archived conferences were infrequently accessed throughout the observation period. Encouraging asynchronous viewing may increase consumption of MGR among faculty who are unable to do so in real time. Offering attendance credit for viewing MGR asynchronously could incentivize otherwise nonattending faculty.

### Limitations

This study did not use surveys or other methods of obtaining feedback from faculty regarding their attendance patterns relative to the mode of MGR presentation, as collecting these data was not feasible given the study’s retrospective design.

Attendance does not guarantee the observer has learned from MGR, although mandatory evaluations may not assess this objective either [10].

### Conclusions

Overall faculty attendance at MGR was neither durably affected by a pandemic-related transition from in-person to a remote format nor by concurrent COVID-19 burden, although individual attendance behaviors varied considerably. If coupled with archived conference recordings, the remote format may be an equally attended and more cost-effective option for presenting MGR in a postpandemic era.

## Abbreviations

CC: critical care
CME: continuing medical education
DOM: Department of Medicine
GIM: general internal medicine
ICU: intensive care unit
ID: infectious diseases
MGR: medical grand rounds

## References

[1] Osler W (1901). The natural method of teaching the subject of medicine. JAMA.

[2] Jattan A, Francois J (2022). Twelve tips for adapting grand rounds for contemporary demands. Med Teach.

[3] Mueller PS, Litin SC, Sowden ML, Habermann TM, LaRusso NF (2003). Strategies for improving attendance at medical grand rounds at an Academic Medical Center. Mayo Clin Proc.

[4] Yang AZ, Hyland CJ, Xiang DH, Helliwell LA, Broyles JM (2023). Improving the quality of grand rounds in plastic surgery: in-person, hybrid, or virtual. Plast Reconstr Surg Glob Open.

[5] Reddy GB, Ortega M, Dodds SD, Brown MD (2022). Virtual versus in-person grand rounds in orthopaedics: a framework for implementation and participant-reported outcomes. J Am Acad Orthop Surg Glob Res Rev.

[6] Crossman M, Papanagnou D, Sullivan T, Zhang XC (2021). Virtual grand rounds in COVID-19: a financial analysis. Acad Emerg Med.

[7] Monahan S, Monahan K (2023). The potential environmental impact of external speakers' airplane travel to grand rounds conferences. Environ Health.

[8] Sparkes D, Leong C, Sharrocks K, Wilson M, Moore E, Matheson NJ (2021). Rebooting medical education with virtual grand rounds during the COVID-19 pandemic. Future Healthc J.

[9] Dugani SB, Geyer HL, Maniaci MJ, Fischer KM, Croghan IT, Burton C (2021). Psychological wellness of internal medicine hospitalists during the COVID-19 pandemic. Hosp Pract (1995).

[10] Wecksell M, Salik I (2022). Mandatory grand rounds evaluations: more data, less information. Cureus.

